# Supplementary material for: Performance of the Photosynthetic Apparatus under Glass with a Luminophore Modifying Red-To-Far-Red-Light Ratio—A Case Study
Source: Cells. 2023 Jun 5;12(11):1552. doi: 10.3390/cells12111552 (PMC10252551; doi:10.3390/cells12111552)
Supplement: Supplementary file 1 [file cells-12-01552-s001.zip › cells-2102307 supplementary material.pdf]

Supplementary Materials

Table S1. Raw values of structural and functional parameters of photosynthetic apparatus of butterhead and iceberg lettuce types cultivated in transparent (control) and red glasshouses.

Parameter	Butterhead		Iceberg	
	Control	Red	Control	Red
Area	82982±4205	39432*±6704	67170±1132	38485*±5575
F_0	466±15	407*±9	440±5	387*±13
Fm	3657±206	3110*±177	3382±152	2900*±81
Fv	3191±221	2703*±185	2942±156	2513*±78
Fv/Fm	0.87±0.02	0.87±0.01	0.87±0.01	0.87±0.00
Fv/F_0	6.85±0.80	6.65±0.60	6.69±0.43	6.52±0.23
V_J	0.44±0.00	0.50*±0.01	0.48±0.01	0.52*±0.01
V_I	0.76±0.00	0.89*±0.02	0.80±0.01	0.87*±0.00
φPo	0.83±0.02	0.80*±0.01	0.81±0.01	0.78*±0.00
φEo	0.46±0.01	0.40*±0.01	0.42±0.01	0.37*±0.01
Sm	27.4±0.5	15.9*±2.5	24.4±0.9	16.9*±1.6
ABS/RC	2.57±0.16	3.31*±0.12	2.86±0.06	3.66*±0.15
TRo/RC	2.04±0.09	2.67*±0.08	2.27±0.03	3.00*±0.13
ETo/RC	1.14±0.05	1.34*±0.05	1.18±0.03	1.43*±0.06
DIo/RC	0.33±0.08	0.44*±0.05	0.37±0.03	0.49*±0.02
RC/CSo	256±8	187*±9	224±6	163*±17
TRo/CSo	519±10	500*±7	510±5	490*±2
ETo/CSo	290±3	250*±5	264±7	234*±5
DIo/CSo	107±10	126*±7	116±5	136*±2
ψEo	0.56±0.00	0.50*±0.01	0.52±0.01	0.48*±0.01
ϱRo	0.24±0.00	0.11*±0.02	0.20±0.01	0.13*±0.00
δRo	0.43±0.01	0.23*±0.04	0.38±0.02	0.28*±0.00
φRo	0.20±0.00	0.09*±0.02	0.16±0.01	0.11*±0.00

* statistically significant difference within each parameter and lettuce type at $p \leq 0.05$; (n = 10)

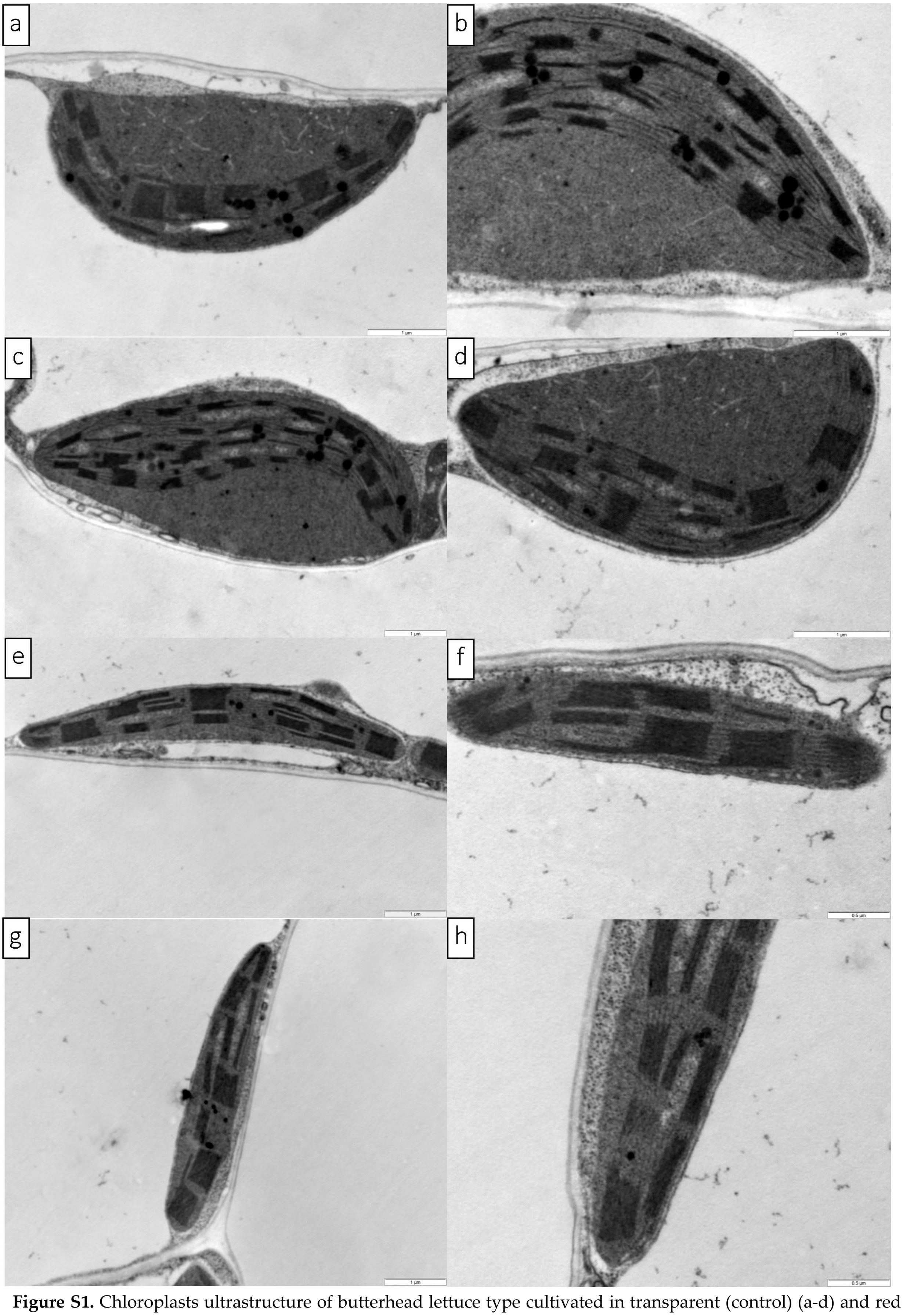

Figure S1. Chloroplasts ultrastructure of butterhead lettuce type cultivated in transparent (control) (a-d) and red (e-f) glasshouses. Scale bars: 1 μm (a-d, e,g) 0.5 μm (f,h) .

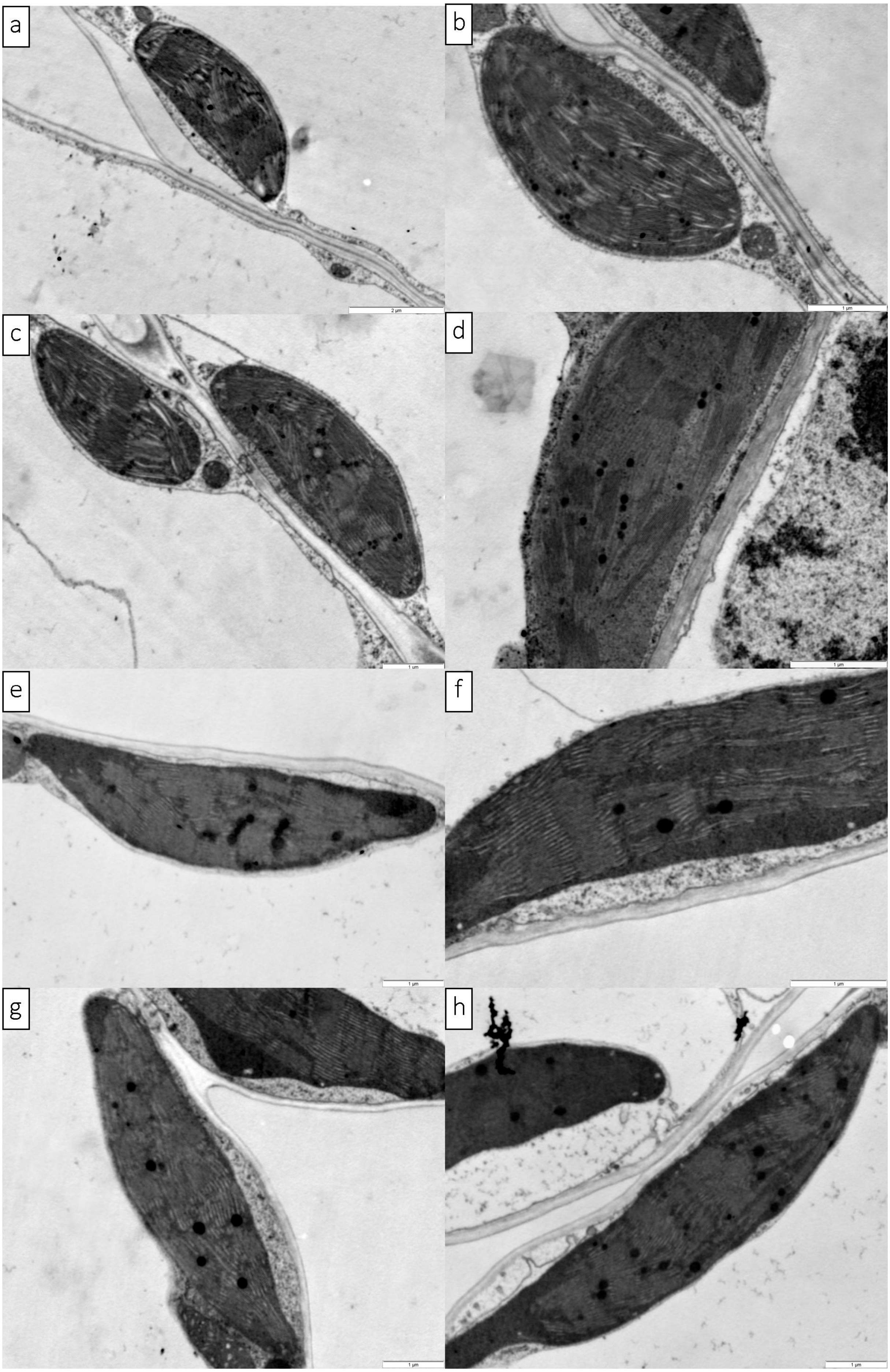

Figure S2. Chloroplasts ultrastructure of iceberg lettuce type cultivated in transparent (control) (a-d) and red (e-f) glasshouses. Scale bars: 1 μm

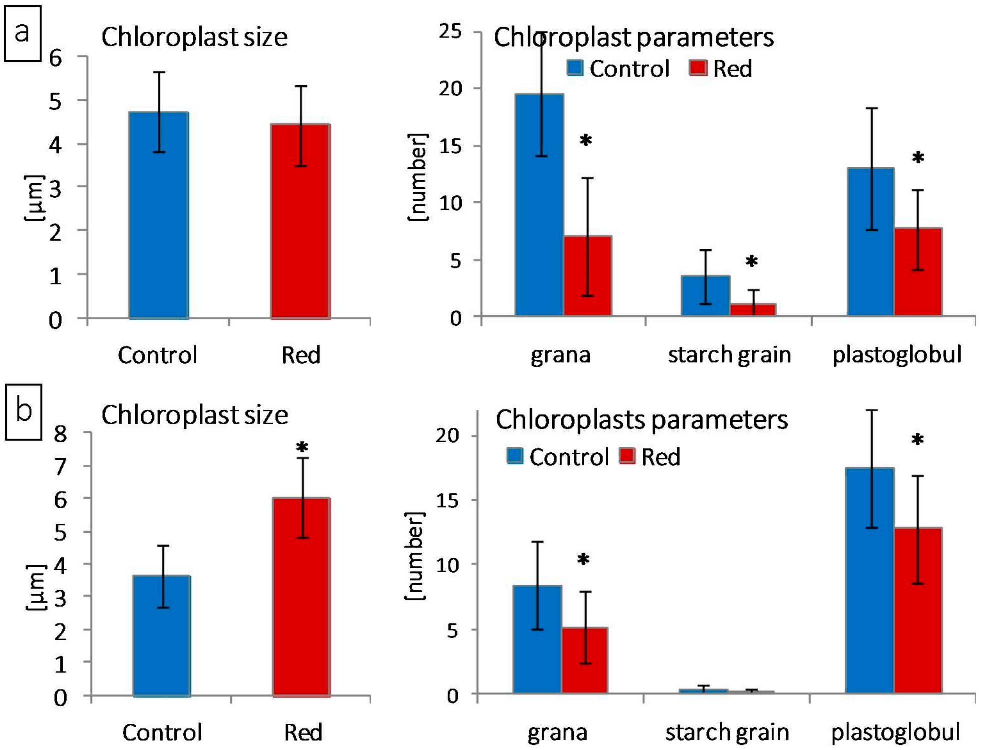

Figure S3. Chloroplasts size and parameters (number of grana, starch grains and plastoglobuli) of butterhead (a) and iceberg (b) lettuce type cultivated in transparent (control) and red glasshouses. * statistically significant difference within each parameter at $p \leq 0.05$; ($n = 10$)
